# A brain functional network framework based on EEG connectivity for major depressive disorder recognition

**DOI:** 10.3389/fnhum.2026.1930925

**Published:** 2026-10-07

**Authors:** Min Fang, Tian-ao Cao, Jionghang Wu, Huixin Liu, Jijun Tong, Zhengkui Chen

**Affiliations:** 1 School of Computer Science and Technology, Zhejiang Sci-Tech University, Hangzhou, China; 2 School of Information Science and Engineering, Zhejiang Sci-Tech University, Hangzhou, China

**Keywords:** major depressive disorder, EEG, brain functional network, phase-locking value, Pearson correlation coefficient

## Abstract

**Introduction:**

When the brain is regarded as a complex dynamic system, its abnormalities often show the overall network reorganization. Traditional analysis for major depressive disorder (MDD) based on single-channel features of electroencephalogram (EEG) mainly focuses on signal changes, while ignoring inter-regional functional connectivity and network topology. As a consequence, it is challenging to reveal the systemic abnormal mechanisms.

**Methods:**

To address this problem, this study built a framework of multi-frequency brain functional network (BFN) construction and recognition based on resting-state EEG. Phase-locking value (PLV) and Pearson correlation coefficient (PCC) were separately utilized to characterize phase synchronization and amplitude correlation. Abnormal BFNs in MDD were systematically evaluated from both connectivity patterns and node topology.

**Results:**

Results suggest that MDD patients exhibit increased connectivity in the left frontal, central, and parietal regions in the low-frequency bands. In the mid-frequency bands, increased fronto-central connectivity and reduced posterior connectivity are observed. In the high-frequency band, the connectivity pattern is characterized by increased fronto-temporal and centro-temporal connectivity, together with reduced connectivity in the posterior regions. At the node level, the most prominent alterations are concentrated in the parietal region, particularly in the α band, where altered betweenness centrality (BC) is consistently identified across both PLV and PCC networks. On this basis, the support vector machine (SVM) classifier was employed to evaluate the discriminative capability of PLV-Edge, PCC-Edge, PLV-Node, and PCC-Node features for MDD identification. Among the four feature types, PLV-Node features achieve the highest mean classification balanced accuracy of 81.44%, and PCC-Node features achieve a mean balanced accuracy of 79.14%.

**Discussion:**

All four feature types demonstrate discriminative potential for distinguishing patients with MDD from healthy controls (HCs). These findings suggest that functional connectivity-based brain network features may provide meaningful information for the auxiliary identification of MDD.

## 1 Introduction

Major depressive disorder (MDD) is a common and severe mental disorder with an accelerating global spread. The World Health Organization (WHO) disclosed there are approximately 350 million MDD patients (Lu et al., 2021). Each year, about 850,000 suicides are attributed to MDD (Zhang et al., 2019), accounting for 53.7% of all suicides (Zhang et al., 2020a). Given the high morbidity and mortality of MDD, it is essential to explore its underlying neural mechanisms for more effective diagnosis and further treatment.

Currently, questionnaire-based assessment is the most common method in the clinical diagnosis of depression. However, this method relies heavily on patients' statements and clinicians' experience. It is difficult to establish objective, precise, and quantifiable diagnostic criteria. The development of neuroimaging technologies, such as electroencephalogram (EEG), magnetoencephalography (MEG) and functional magnetic resonance imaging (fMRI), has provided new ideas. Among these, EEG has been widely applied in investigating the pathological mechanisms of depression and developing diagnostic models because of its easy accessibility, high temporal resolution and low cost (Jaseja, 2023).

In traditional EEG-based studies for MDD, researchers focus on extracting time-domain and frequency-domain features to explore abnormalities. For example, information of temporal and frequency-domain was integrated to improve classification performance (Gan et al., 2025). Frequency-domain features such as band power and hemispheric asymmetry across brain regions were made use of (Liu et al., 2024). However, these methods rely on features derived from single or multiple EEG channels and fail to regard the brain as an integrated dynamic system, resulting in inherent limitations.

With the popularity of graph theory, new approaches have been presented for the construction and analysis of brain functional networks (BFNs) (Yu et al., 2023). Through quantitative analysis of topological metrics, the characteristics of BFNs can be effectively described. Numerous studies have demonstrated that MDD is associated with abnormal BFN structures (Hasanzadeh et al., 2020). BFN analysis based on graph theory enables the characterization of the pathological features of MDD, providing a theoretical foundation for elucidating neuropathological mechanisms and exploring potential biomarkers.

## 2 Related works

### 2.1 EEG-based brain network construction in MDD

Constructing BFNs based on EEG involves quantifying the relationships between neural activities from electrodes. Synchronization and correlation are two key dimensions of functional connectivity. Synchronization characterizes the degree of phase coordination among signals and correlation measures the relationship between signal amplitudes (Zhang et al., 2020b).

Synchronization reflects the coordinated activity between brain regions or nodes. Networks were constructed based on synchronization likelihood (SL) for 12 MDD patients and 16 healthy controls (HCs) (Park et al., 2007). The results revealed a significant reduction of SL in the δ (0.5–4 Hz) band in the MDD group. The phase synchronization index (PSI) was calculated to construct BFNs. Results displayed the number of significantly synchronized electrode pairs diminished in the δ band but grew in the θ (4–8 Hz), α (8–13 Hz), and β (13–30 Hz) bands in MDD patients (Li et al., 2016). BFNs were constructed based on the phase lag index (PLI) and topological changes across frequency bands and brain regions were analyzed (Zhang et al., 2020b). The results implied that power spectral density in the θ and α2 bands rose in MDD patients. PLV networks were constructed in the θ and α bands (Shen et al., 2020). Network strength, average characteristic path length, and average clustering coefficient were used as features and fed into support vector machine (SVM) classifier. The results illustrated that the performance of the three network properties was significantly improved in both bands. In addition, the area under the curve (AUC) of three network properties was also made use of as features for classification. Using the AUC of network properties as features in the α band improved the classification accuracy by about 10% compared to network parameter features.

Correlation-based BFNs characterize functional connectivity by modeling electrodes as nodes and their linear correlations as edges. In a previous study, source localization was performed on 128-channel EEG data and 68 regions of interest (ROIs) were defined as nodes to construct Pearson correlation coefficient (PCC)-BFNs (Lin et al., 2022). Results showed that the clustering coefficient and local efficiency increased significantly in MDD patients. Meanwhile, global efficiency and characteristic path length decreased. Four linear features (power spectral density, activity, mobility, and complexity) were used as node features (Zhu et al., 2022). A learnable weight matrix was introduced to identify important edges and nodes. Results revealed that the temporal lobe and parieto-occipital region had significant effects in depression recognition. PCC-BFNs were constructed for 13 MDD patients and 13 HCs (Zhang et al., 2018). The results indicated a more randomized global network organization in MDD, characterized by higher global efficiency and lower clustering coefficient, characteristic path length, and local efficiency. PCC and mutual information (MI) were integrated to construct a joint functional brain network (PMNet) (Jiang et al., 2023). This integrated the time-domain and frequency-domain features of EEG effectively, enhancing the expressive capability of BFN features.

Current studies demonstrate that constructing BFNs based on EEG is able to effectively characterize topological abnormalities in depression. These abnormal features are considered potential biomarkers for objective identification and auxiliary diagnosis of MDD.

### 2.2 Feature selection of brain functional networks

EEG is typically acquired using 64 or 128 electrodes. When electrodes are treated as nodes and functional connectivity is considered as edges, BFNs are represented as high-dimensional feature matrices. The feature dimension increases quadratically with the number of nodes. For an undirected network with *N* nodes, the number of edges can reach *N*(*N*−1)/2. Feature dimensions further increase when multiple frequency bands and connectivity measures are considered. High-dimensional features greatly increase the computational cost of model training and parameter optimization. They may also introduce redundant information and cause classifier overfitting. Therefore, feature selection is essential for improving the robustness, interpretability, and clinical applicability of BFN analysis.

Existing studies mainly address this issue using three different strategies. The first strategy simplifies hardware or data acquisition at the channel level. This method directly reduces the number of EEG channels and the network size at the source. The second strategy focuses on feature-level ranking and selection. It retains the most discriminative subsets from numerous connectivity features. The third strategy uses structural aggregation and representation learning methods such as graph-theoretical metrics, modular features, and group-level fusion networks. These methods replace original fully connected matrices with lower-dimensional and physiologically meaningful representations. These approaches aim at preserving useful information while removing redundancy and suppressing noise. They also aim to improve the stability and interpretability of network features.

Four EEG configurations were designed with 62, 30, 19, and 10 channels to evaluate the effect of channel reduction on MDD classification (Shim et al., 2023). The results showed that reducing channels from 62 to 19 caused no significant decrease in classification performance. This suggests that not all EEG channels contribute equally to disease identification. Besides fixed channel reduction, some studies adopted automatic channel selection methods. For example, statistical feature ranking was combined with SVM to evaluate EEG channel importance (Acharya et al., 2018). Results manifested that frontal and frontocentral regions contributed significantly more to depression recognition. In addition, some studies applied intelligent optimization algorithms, such as genetic algorithm (GA) and particle swarm optimization (PSO), to search for optimal channel combinations (Alotaiby et al., 2014). These studies indicate that MDD-related abnormalities are mainly concentrated in specific brain regions. Therefore, channel selection based on prior brain knowledge or optimization algorithms is an important strategy for feature extraction.

Apart from directly reducing node numbers, a more common strategy is feature ranking and selection for brain networks. Researchers have employed filter, wrapper, and embedded feature selection methods to identify discriminative connectivity subsets from high-dimensional feature spaces. A two-stage feature selection framework was proposed, combining PCC and recursive feature elimination (RFE) (Li et al., 2022). First, PCC was used to quickly remove features weakly correlated with class labels and reduce the search space. Then, RFE recursively eliminated redundant features from the remaining subset through supervised learning. Finally, the optimal discriminative feature combination was obtained. This framework combined the high efficiency of filter methods with the strong classification ability of wrapper methods. The results demonstrated that staged feature compression effectively reduced redundant connections in high-dimensional EEG features. Meanwhile, some studies incorporated attention mechanisms from deep learning to automatically learn important connectivity weights through network training (Ying et al., 2024). In addition, differential connectivity analysis is another important feature selection strategy for BFNs. Researchers usually apply *t*-tests, Wilcoxon rank-sum tests, or network permutation tests to identify significant connectivity differences between MDD patients and HC subjects. Significant connections are then retained as classification features. This method could reduce feature dimensions while maintaining strong physiological interpretability (Kaiser et al., 2015).

With the development of complex network theory, researchers have attempted to use graph metrics, modular features, and fusion networks for high-level representation of complex connectivity patterns. These methods are able to reduce feature dimensions and improve the stability of network features. A clustering-fusion feature selection method was proposed to address depression heterogeneity (Sun et al., 2023). First, hierarchical clustering was applied to identify potential heterogeneous distributions among subjects. Then, average fusion and similarity network fusion (SNF) were used to construct BFN maps for different subgroups. Finally, discriminative features were extracted through differential analysis. The results showed that this method outperformed traditional feature selection approaches at both sensor and source levels. In particular, classification performance in the β band improved by more than 6%. Besides fusion networks, graph-theoretical metrics are also important structural aggregation methods. Researchers commonly make use of clustering coefficient, characteristic path length, global efficiency, and small-worldness to compress high-dimensional connectivity matrices (Rubinov and Sporns, 2010). Compared with using all connectivity edges directly, these graph metrics could characterize brain network organization in terms of network integration and segregation. They also provided clearer neurophysiological interpretations.

### 2.3 Deep learning approaches for MDD classification

Deep learning has been widely applied in EEG classification because of its strong modeling capability. Early studies mainly used deep learning models to directly process raw EEG signals. For instance, ScalingNet was proposed to automatically extract hierarchical features from raw EEG signals using convolutional structures (Hu et al., 2021). Convolutional neural network (CNN) and Transformer modules were combined to extract local spatiotemporal features and global dependencies (Xue et al., 2024). In addition, recurrent neural network (RNN) (Kuanar et al., 2018) and long short-term memory network (LSTM) (Delimayanti et al., 2024) were applied to EEG time-series modeling. These models were used to capture dynamic temporal dependencies in EEG signals. According to model architecture, existing methods have gradually developed into different branches. CNN is mainly used to extract local spatial features from EEG signals. CNN employs parameter sharing and local receptive fields to reduce model complexity effectively. Transformer learns long-range dependencies through self-attention mechanisms. Therefore, they are more suitable for capturing global associations between different brain regions. RNN and LSTM focus more on temporal dynamic variations in EEG sequences. Thus, they are suitable for continuous EEG time-series analysis. In recent years, some studies adopt hybrid architectures combining CNN, Transformer, and LSTM. These hybrid models could simultaneously learn spatial, temporal, and global EEG features to improve classification performance.

With the development of BFN, more studies have used functional connectivity matrices as inputs for deep learning models. Functional connectivity networks were constructed using high-density resting-state EEG signals (Sun et al., 2019). They further analyzed abnormal topological characteristics in MDD patients using graph-theoretical metrics. Betweenness centrality (BC) in the right temporal region of the α band showed the best performance. The classification accuracy reached 87.50%. Compared with directly using raw EEG signals, BFNs were competent to better reflect cooperative interactions between brain regions. Thence, BFNs could provide improved physiological interpretability.

On this basis, research has gradually shifted from network feature extraction to network structure learning. Recently, graph neural network (GNN) has been increasingly applied to BFN analysis. A dynamical graph convolutional neural network (DGCNN) was presented (Song et al., 2018). This model learned connectivity relationships in BFNs through graph convolution operations. It also enabled automatic extraction of EEG graph-structured features. Unlike traditional CNNs, GNNs were capable of processing non-Euclidean graph-structured data directly. Hence, they were more suitable for BFN modeling. Additionally, a graph attention network (GAT) (Veličković et al., 2017) adaptively learned the importance of different connectivity edges through attention mechanisms. This strategy could enhance the representational ability of critical pathological connections.

Overall, EEG-based deep learning methods have gradually evolved from raw signal modeling to multi-level modeling frameworks. These frameworks integrate spatiotemporal features, BFNs, and graph structure learning. This tendency not only improves MDD classification performance but also provides new insights into abnormal brain network mechanisms in depression.

## 3 Materials and methods

Figure 1 shows the schematic diagram of the proposed method. The simplified process is as follows: (1) Resting-state EEG data from the Multi-modal Open Dataset for Mental-disorder Analysis (MODMA) Dataset were selected. (2) EEG signals were preprocessed. (3) BFNs were constructed based on PLV and PCC. (4) The constructed networks were thresholded. (5) Edge and node features were extracted to identify potential biomarkers with significant differences. (6) Classification of MDD and HC.

**Figure 1:**
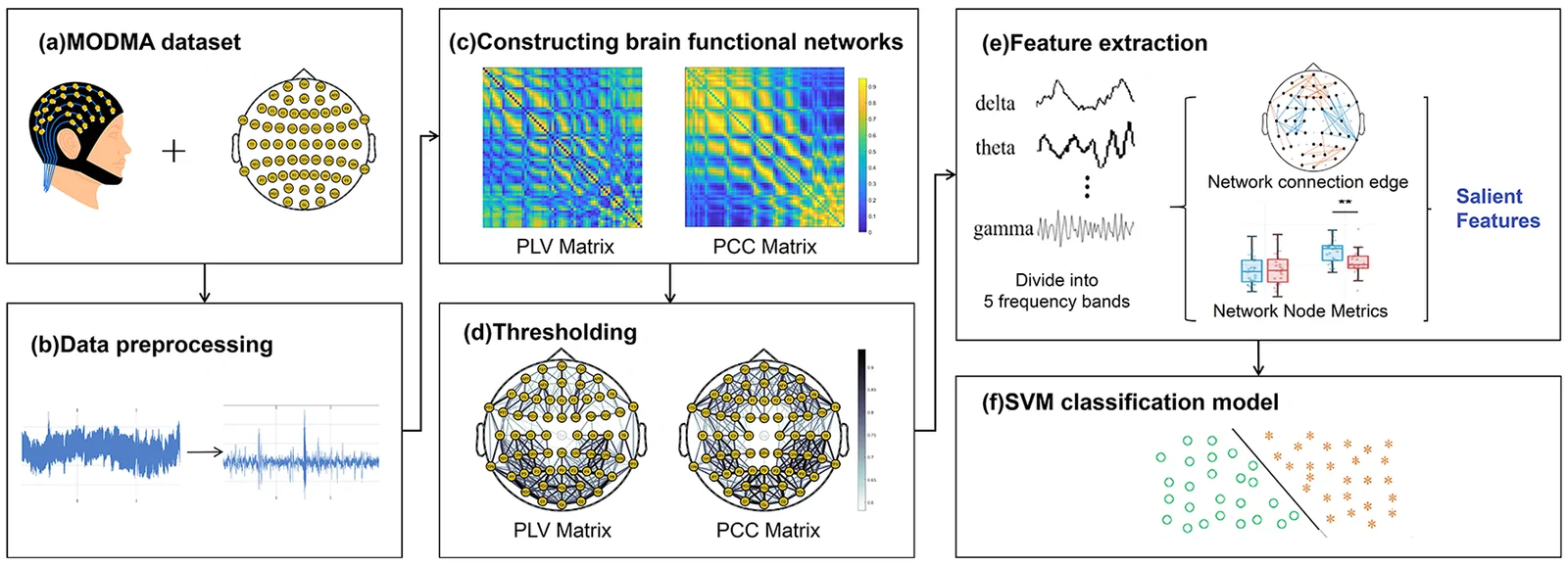
The schematic diagram of the proposed method. **(a)** MODMA dataset. **(b)** Data processing. **(c)** Constructing brain functional networks. **(d)** Thresholding. **(e)** Feature extraction. **(f)** SVM classification model.

### 3.1 Experimental paradigm and preprocessing

We used the 128-channel resting-state EEG subset from the publicly available MODMA Dataset. The subset comprises the EEG signals of 24 MDD patients and 29 HCs, with their basic information summarized in Table 1. Before enrollment, age and sex were collected for all participants. Participants also completed the PHQ-9 self-report questionnaire. Exclusion criteria included organic brain damage, severe physical illness, substance dependence (alcohol or psychotropic drugs), pregnancy, and lactation. All the MDD patients were recruited from the outpatient department of the Psychiatry Department of Lanzhou University Second Hospital. MDD patients were clinically diagnosed by psychiatrists and further assessed using the structured Mini-International Neuropsychiatric Interview (MINI) based on Diagnostic and Statistical Manual of Mental Disorders, Fourth Edition (DSM-IV) diagnostic criteria. In addition, MDD patients received no psychotropic drug treatment within 2 weeks before the experiment and had a PHQ-9 score of five or higher. Healthy controls were recruited via posters. HCs were selected using strict exclusion criteria: no diagnosis of any mental disorder based on the MINI, no personal or family history of psychiatric illness, and absence of depression-related clinical symptoms (Cai et al., 2022).

**Table 1:** Dataset information.

Sex	11 female		13 male	
Mean age	27.54 ± 9.13	33.69 ± 10.85	9 female	20 male
Age range	18–45	16–52	29.00 ± 7.84	32.55 ± 9.65
PHQ-9 score	19.00 ± 4.40	17.76 ± 2.55	21–41	19–52

The PHQ-9 is an internationally used self-assessment scale for depressive symptoms. It consists of nine questions, with a total score ranging from 0 to 27. Participant labels were established based on the clinical diagnosis made by physicians. PHQ-9 scores were collected as complementary quantitative information for characterizing depressive symptoms and were not used as the basis for participant grouping.

To further describe the characteristics of the subjects, Figure 2 shows a comparison of age and sex distributions between the full MDD and HC groups. Age was compared using Wilcoxon rank-sum test, and sex distribution was assessed using Fisher's exact test. The MDD and HC groups are comparable in terms of age and sex distributions.

**Figure 2:**
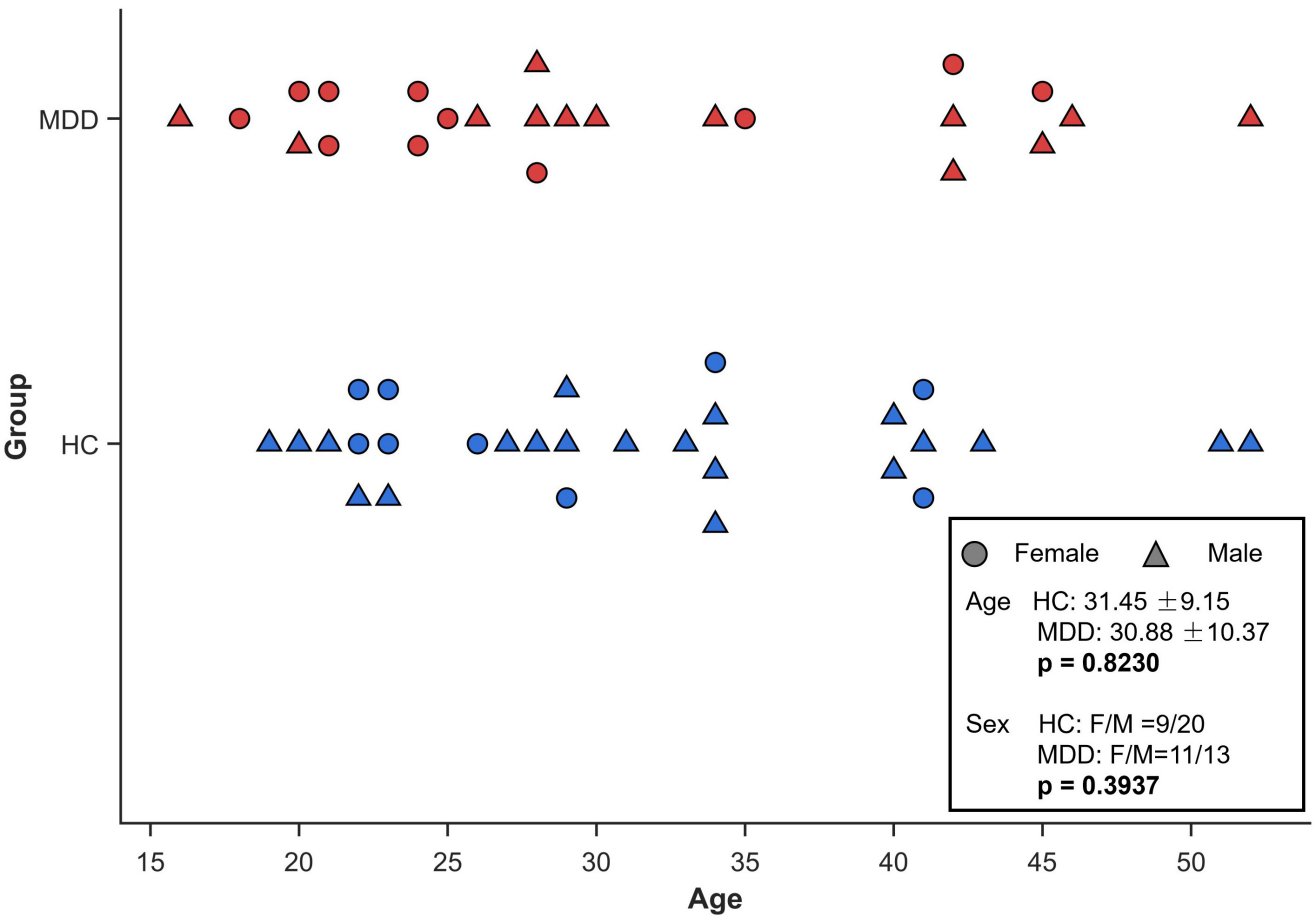
Age and sex distributions of MDD and HC groups.

For each participant, 5 min of EEG data were recorded at the sampling frequency of 250 Hz. Data were collected in a quiet, sound-attenuated environment without strong electromagnetic interference. A 128-channel HydroCel Geodesic Sensor Net EEG cap and Net Station 4.5.4 software were used. All electrode impedances were kept below 50 kΩ prior to data acquisition. Participants sat comfortably with eyes closed, awake and motionless.

Then we preprocessed the EEG to improve signal quality. First, a 0.5–48 Hz, 249-order finite impulse response (FIR) bandpass filter was applied to the raw EEG signals to suppress low-frequency drift, high-frequency noise, and powerline interference of 50 Hz. The FIR filter was designed using a Hamming window and applied with zero-phase filtering to minimize any additional impact on the phase information of EEG. Next, independent component analysis (ICA) was performed using the Extended Infomax algorithm in the EEGLAB toolbox, and ICA components were automatically classified using ICLabel. Any ICA component with an Eye probability ≥0.80 or Muscle probability ≥0.80 was removed.

To reduce network scale and computational complexity, we selected 64 channels for BFN construction. As shown in Figure 3, the spatial distribution of the 64 channels and the reference electrode Cz is presented. Cz is not classified within the brain regions and does not serve as a channel for constructing the brain network.

**Figure 3:**
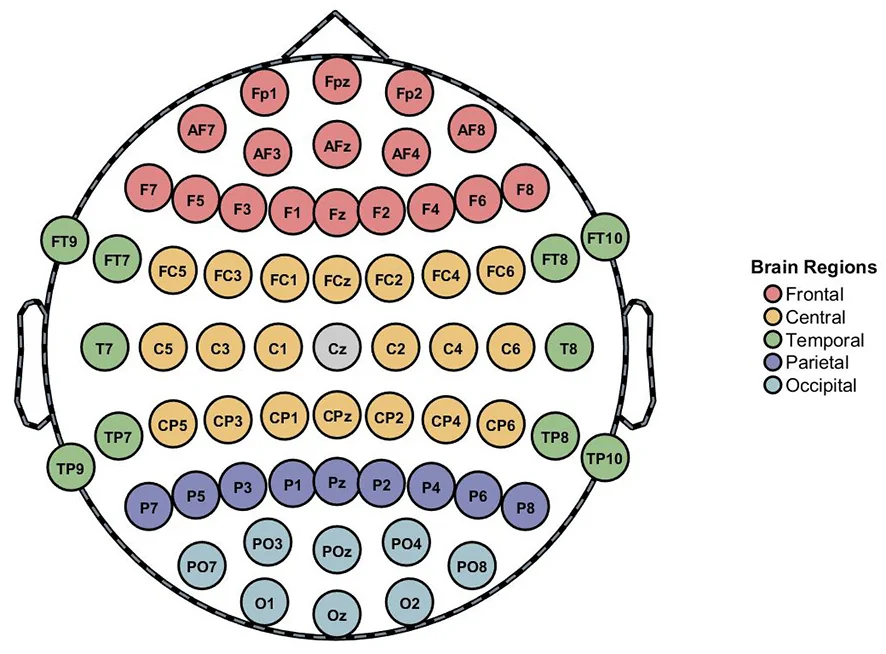
65-channel position distribution.

Each participant has 300 s of continuous EEG data. To reduce potential instability at the beginning and the end of the data, we took the 290 s of EEG signals from 5 to 295 s for further analysis. The selected EEG signal was divided into five frequency bands: δ (0.5–4 Hz), θ (4–8 Hz), α (8–13 Hz), β (13–30 Hz), and γ (30–48 Hz), for subsequent frequency-specific brain functional network construction and analysis. Finally, the continuous EEG signal was segmented into 4-s time windows with 50% overlap between adjacent windows to create EEG segments for subsequent training and testing of classification model.

### 3.2 Construction of brain functional networks

In this study, PLV and PCC were utilized to calculate the connection strength between nodes. PLV measures the phase synchronization between nodes, reflecting the relative temporal consistency of neural activity (Wang et al., 2020; Zhao et al., 2026). PCC measures the linear correlation between nodes (Moon et al., 2020). The combination of the two measures constructs a multilayer BFN reflecting non-linear phase synchronization and linear amplitude correlation. This enables a comprehensive representation of brain connectivity features. PLV and PCC calculated at the scalp level could both be affected by volume conduction, common references, and spatial mixing. Hence, the connectivity abnormalities found in this study reflect changes in functional coupling at the scalp level.

PLV is defined as the absolute value of the mean phase difference between two nodes. The formula is listed in Equation 1:


$$\begin{array}{c} PLV\left(t\right)=\frac{1}{M}\left|\overset{M}{\underset{n=1}{\sum }}\exp\left(j\left(\Delta \varphi_{n}\left(t\right)\right)\right)\right| \end{array}$$ (1)

where Δφ_*n*_(*t*) = φ_*x*_(*t*)−φ_*y*_(*t*) represents the phase difference between electrodes *x* and *y* at time *t*. *M* denotes the length of the time series. A value of PLV ranges from 0 to 1. The value of 0 indicates no phase synchronization between the two signals, while 1 indicates complete phase synchronization.

PCC quantifies the linear relationship between two EEG sequences (Ahmadi Moghadam et al., 2024). The formula is shown in Equation 2:


$$\begin{array}{c} PCC_{xy}=\frac{\overset{M}{\underset{t=1}{\sum }}\left(x_{t}-\bar{x}\right)\left(y_{t}-\bar{y}\right)}{\sqrt{\overset{M}{\underset{t=1}{\sum }}{\left(x_{t}-\bar{x}\right)}^{2}}\sqrt{\overset{M}{\underset{t=1}{\sum }}{\left(y_{t}-\bar{y}\right)}^{2}}} \end{array}$$ (2)

where *x*_*t*_ and *y*_*t*_ denote the signal values of channels *x* and *y* at time point *t*, respectively. $\bar{x}$ and $\bar{y}$ represent the mean values within the current time segment. When the correlation between two variables is strong, the correlation coefficient approaches 1 or −1. The correlation coefficient equaling 0 indicates no correlation between them. The sign reflects the variation trend of activity between two nodes. A positive correlation indicates simultaneous increase or decrease, whereas a negative correlation indicates opposite changes.

### 3.3 Threshold of brain functional networks

BFNs contain a large number of weak and spurious connections which weaken the core characteristics. To solve this problem, these links can be removed through thresholding. Specifically, network sparsity is used as the threshold criterion, retaining connections with higher weights and setting weaker connections to zero. Sparsity is defined as the ratio of the actual number of edges in the network to the maximum possible number of edges.

For the PCC network, the absolute values of the PCCs were used as connection strengths to rank inter-channel connections before thresholding. The corresponding proportion of connections was retained according to the predefined network sparsity. Specifically, taking the absolute value preserves the magnitude of the correlation but discards its sign. Therefore, the resulting network represents the strength of statistical association between EEG channels rather than the direction of the correlation. The absolute PCC was taken advantage of as the network connection indicator (Klepl et al., 2022). The absolute value of the PCC was used to represent the strength of EEG functional connectivity (Xu et al., 2023). Methodological research has shown that using absolute values rather than only positive or negative coefficients can improve the test-retest reliability of global graph-theoretical measures. This approach preserves the strength of both positive and negative neural coupling while avoiding potential bias caused by inter-subject differences in the proportions of positive and negative connections (Ran et al., 2020).

**•Shortest path length**: The shortest path *L*_*ij*_ is the path with the minimum sum of edge lengths between nodes *i* and *j*. In weighted networks, the edge length is the reciprocal of the edge weight, as shown in Equation 3:


$$\begin{array}{c} l_{ij}\text{=}\frac{\text{1}}{w_{ij}} \end{array}$$ (3)

where *l*_*ij*_ denotes the length of the edge between nodes *i* and *j*, and *w*_*ij*_ denotes the connection weight between nodes *i* and *j*. The average shortest path length of a weighted network is defined as Equation 4:


$$\begin{array}{c} L_{w}\text{=}\frac{\text{1}}{N\text{(}N\text{-1)}}\sum_{i=1,i\neq j}^{N}L_{ij} \end{array}$$ (4)

where *N* is on behalf of the number of nodes in the network.

**•Clustering coefficient**: The clustering coefficient *C*_*i*_ is used to measure the closeness of nodes in a network tend to cluster together. It reflects the likelihood that two neighbors of a node are also connected to each other, as listed in Equation 5:


$$\begin{array}{c} C_{i}\text{=}\frac{\underset{j,k,j\neq k}{\sum }w_{ij}w_{ik}w_{jk}}{\underset{j,k,j\neq k}{\sum }w_{ij}w_{ik}} \end{array}$$ (5)

where *j* and *k* are nodes adjacent to *i, w* represents the connection weight between nodes. The average clustering coefficient of a weighted network is defined as the mean of the local clustering coefficients across all nodes, calculated as Equation 6:


$$\begin{array}{c} C_{w}\text{=}\frac{1}{N}\sum_{i=1}^{N}C_{i} \end{array}$$ (6)

**•** **Small-world index**: The small-world network is characterized by the high clustering coefficient and short average path length. Taking random networks as the reference, it can be defined as Equation 7:


$$\begin{array}{c} \sigma =\frac{C_{w}/C_{\text{rand}}}{L_{w}/L_{\text{rand}}} \end{array}$$ (7)

where *C*_rand_ and *L*_rand_ represent the average clustering coefficient and average shortest path length of 50 random networks with the same size as the original network.

BFNs have small-world properties (Liao et al., 2017) that require the network sparsity to be less than 50%. According to random graph theory, sparsity should exceed $\frac{2\ln\;N}{N}$ to ensure the absence of isolated nodes in the network (Kitzbichler et al., 2011). In this study, we set the sparsity range S∈[0.13, 0.50]. Within this range, we observed the variation of the small-world coefficient σ across network sparsity. As shown in Figure 4, σ gradually increases when sparsity rises. Both PCC and PLV networks simultaneously satisfy the small-world criterion (σ > 1) when *S* exceeds 0.22. Among the sparsity levels satisfying this criterion, *S* = 0.24 was selected as a relatively low network density that provided a small margin above the σ = 1 criterion (Bassett and Bullmore, 2017) for both network types while retaining fewer connections. The network threshold selection was performed independently of the subsequent classification process. This strategy preserves the small-world topology while reducing unnecessary network complexity. Exactly, this value represents a practical threshold chosen to balance small-world topology and network sparsity.

**Figure 4:**
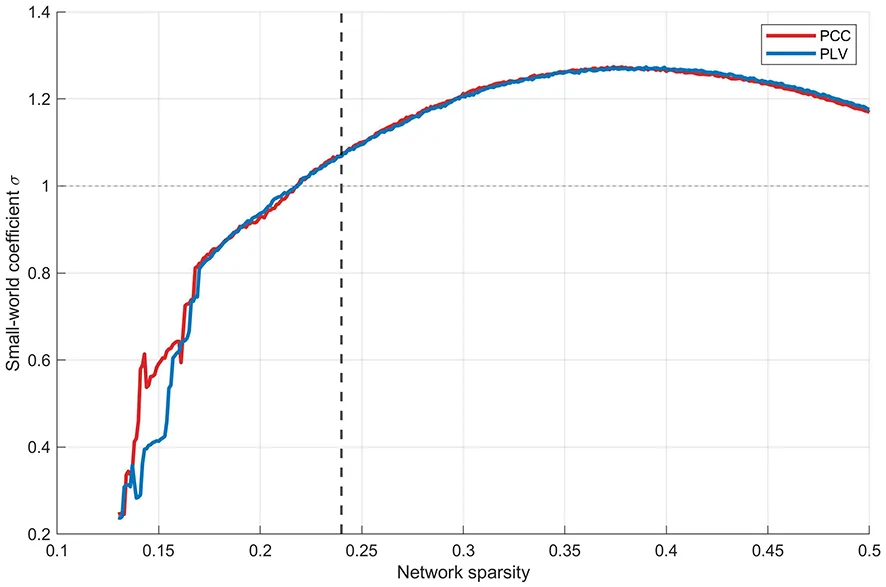
Variation of the small-world coefficient (σ) of PCC and PLV brain networks across network sparsity. The curves represent the mean σ across participants.

To assess the robustness of network metrics with changes in network sparsity, Cohen's d was used as a standardized effect size. The graph-theoretical measures analyzed in this study included betweenness centrality (BC), local efficiency (E_loc_), and clustering coefficient (CC). Cohen's *d* was calculated as the difference between the two group means divided by their pooled standard deviation, as displayed in Equation 8:


$$\begin{array}{c} d=\frac{{\bar{X}}_{\text{MDD}}-{\bar{X}}_{\text{HC}}}{S_{p}} \end{array}$$ (8)

where *d* denotes the Cohen's d effect size; ${\bar{X}}_{\text{MDD}}$ denotes the sample mean of the MDD group; ${\bar{X}}_{\text{HC}}$ means the sample mean of the HC group; and *S*_*p*_ is on behalf of the pooled standard deviation of the MDD and HC groups, calculated as Equation 9:


$$\begin{array}{c} S_{p}=\sqrt{\frac{\left(n_{\text{MDD}}-1\right)S_{\text{MDD}}^{2}+\left(n_{\text{HC}}-1\right)S_{\text{HC}}^{2}}{n_{\text{MDD}}+n_{\text{HC}}-2}} \end{array}$$ (9)

where *n*_MDD_ and *n*_HC_ denote the sample sizes of the MDD and HC groups, respectively, and *S*_MDD_ and *S*_HC_ are their corresponding sample standard deviations. The sign of Cohen's *d* indicates the direction of the between-group difference: a positive *d* indicates a higher value in the MDD group than in the HC group, whereas a negative *d* indicates a lower value in the MDD group. A larger absolute value of Cohen's *d* indicates a greater standardized between-group difference.

In addition, the two-sided Wilcoxon rank-sum test was used to assess between-group differences in BC, E_loc_, and CC between the MDD and HC groups. The corresponding *p* values were reported together with Cohen's *d*. Cohen's *d* (*p*) in the Table 2 represents the standardized between-group effect size and the corresponding *p* value from the Wilcoxon rank-sum test, respectively. As demonstrated in Table 2, three node metrics maintain consistent directions of between-group differences and generally comparable effect sizes across the nearby sparsities (*S* = 0.22, 0.23, 0.24, 0.25, and 0.26), indicating reasonably stable to small variations in network sparsity.

**Table 2:** Sensitivity statistics of three node metrics across neighboring network sparsity levels.

Network	Band	Metric	Region	*S*=0.22	*S*=0.23	*S*=0.24	*S*=0.25	*S*=0.26
PLV	δ	BC	Central	0.310 (0.2737)	0.695 (0.0339)	**0.880 (0.0042)**	0.663 (0.0074)	1.011 (0.0018)
PLV	θ	BC	Central	0.756 (0.0082)	0.766 (0.0106)	**0.877 (0.0073)**	0.821 (0.0098)	0.989 (0.0015)
PLV	α	BC	Frontal	−0.894 (0.0025)	−0.938 (0.0032)	**−0.836 (0.0084)**	−0.804 (0.0048)	−0.684 (0.0256)
PLV	α	BC	Central	0.521 (0.0969)	0.763 (0.0174)	**0.665 (0.0192)**	0.623 (0.0536)	0.519 (0.0594)
PLV	α	BC	Parietal	−0.871 (0.0053)	−0.658 (0.0230)	**−0.969 (0.0035)**	−0.871 (0.0056)	−0.680 (0.0259)
PCC	θ	BC	Central	0.558 (0.0237)	0.558 (0.0372)	**0.685 (0.0138)**	0.436 (0.0739)	0.304 (0.1453)
PCC	α	BC	Central	0.759 (0.0125)	0.843 (0.0090)	**0.767 (0.0231)**	0.665 (0.0266)	0.709 (0.0255)
PCC	α	BC	Parietal	−0.596 (0.0612)	−0.774 (0.0119)	**−0.826 (0.0059)**	−0.889 (0.0015)	−0.900 (0.0016)
PCC	α	*E*_loc_	Parietal	0.517 (0.0365)	0.545 (0.0400)	**0.663 (0.0374)**	0.497 (0.1202)	0.507 (0.1039)
PCC	α	CC	Parietal	0.795 (0.0146)	0.681 (0.0202)	**0.746 (0.0036)**	0.994 (0.0013)	1.048 (0.0007)
PCC	β	BC	Parietal	−0.501 (0.0879)	−0.474 (0.1311)	**−0.663 (0.0428)**	−0.483 (0.1004)	−0.526 (0.0754)

Values are presented as Cohen's *d* (*p*). The bold values show the detailed results under the selected threshold (0.24).

### 3.4 Computation of network metrics

Previous studies have demonstrated that the organization of BFNs achieves an effective balance between global information integration and local functional specialization, thereby supporting efficient and stable information processing. Based on complex network theory, the topological properties of BFNs can be quantitatively characterized using metrics. In this study, we calculated three metrics: BC, local efficiency (E_loc_), and clustering coefficient (CC). The definitions and calculations of the three metrics are presented in Table 3. All graph-theoretical analyses were performed in MATLAB using the Brain Connectivity Toolbox (BCT). Finally, we used the Wilcoxon rank-sum test to evaluate the differences in these indicators between MDD and HC. To account for multiple comparisons, the Benjamini-Hochberg false discovery rate (BH-FDR) correction was applied separately to each frequency band metric combination, with the 64 node-wise comparisons within each combination constituting one correction family.

**Table 3:** Formulas and mathematical definitions for network metrics.

Indicator	Formula	Description
Betweenness Centrality (BC)	$BC_{i}=\frac{1}{\left(N-1\right)\left(N-2\right)}\sum_{i\neq j,i\neq h,j\neq h}\frac{\rho_{jh}\left(i\right)}{\rho_{jh}}$	ρ_*jh*_ is the number of shortest path between node *j* and *h*; ρ_*jh*_(*i*) is the number of the shortest paths between nodes *j* and *h* that pass through *i*. *N* is the number of node in the network.
Local Efficiency (E_loc_)	$E_{\text{loc}}=\frac{1}{N}\sum_{i\in \text{V}}E\text{(}i\text{)}$ $=\frac{1}{N}\sum_{i\in {\text{V}}_{i}}\frac{\sum_{j\neq h\in {\text{V}}_{i}}{\left[L_{jh}\left(i\right)\right]}^{-1}}{e_{i}\left(e_{i}-1\right)}$	*E*(*i*) is the local efficiency of node *i*. *V* is the set of nodes in the network. *V_*i*_* is the set of nodes adjacent to node *i*. *L_*jh*_*(*i*) is the shortest path length between neighbors *j* and *h* of node *i*. *e_*i*_* is the number of edges among the neighbors of node *i*.
Clustering Coefficient (CC)	$CC=\frac{1}{N}\sum_{i=1}^{N}C_{i}$	*C_*i*_* is the clustering coefficient of node *i*. *k_*i*_* is the degree of node *i*.

## 4 Results

### 4.1 Significant connectivity edges

To investigate intergroup differences in brain functional network connectivity between MDD patients and healthy controls across δ, θ, α, β, and γ bands, edge-wise Wilcoxon rank-sum tests were performed for all pairwise electrode connections. To control for false positives induced by multiple comparisons, the BH-FDR correction was applied separately within each frequency band, with all pairwise electrode connections within the same frequency band constituting one correction family. Figures 5, 6 indicate the connections with significant differences in five frequency bands. Based on the spatial topological distribution, we summarized the results into three patterns.

- **Low-frequency bands (δ** **and** **θ** **bands)**: Increased connectivity in the left hemisphere

**Figure 5:**
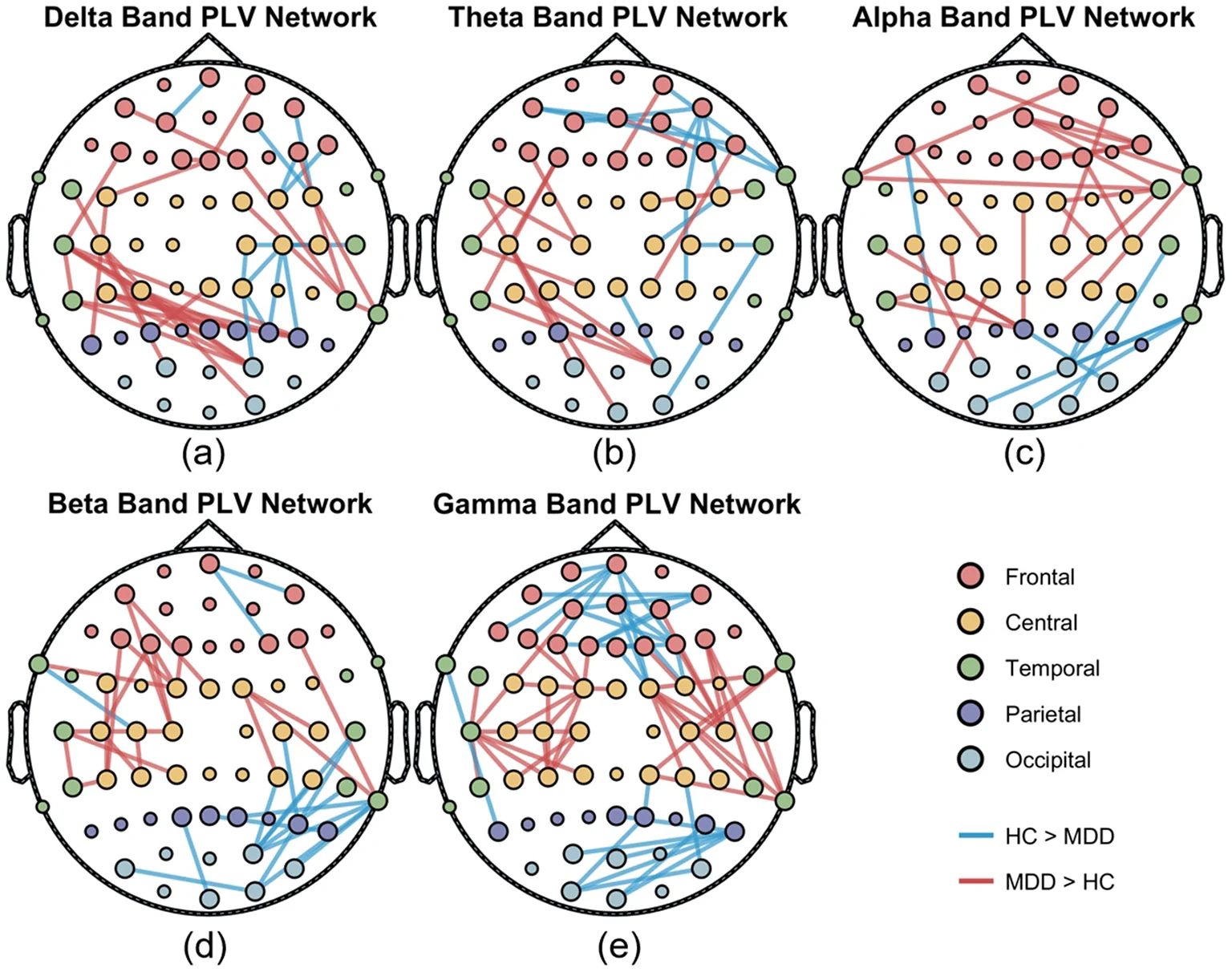
Statistical analysis of the PLV network, illustrating significant connectivity edges (*p* < 0.05) across five frequency bands. **(a)** Delta band. **(b)** Theta band. **(c)** Alpha band. **(d)** Beta band. **(e)** Gamma band.

**Figure 6:**
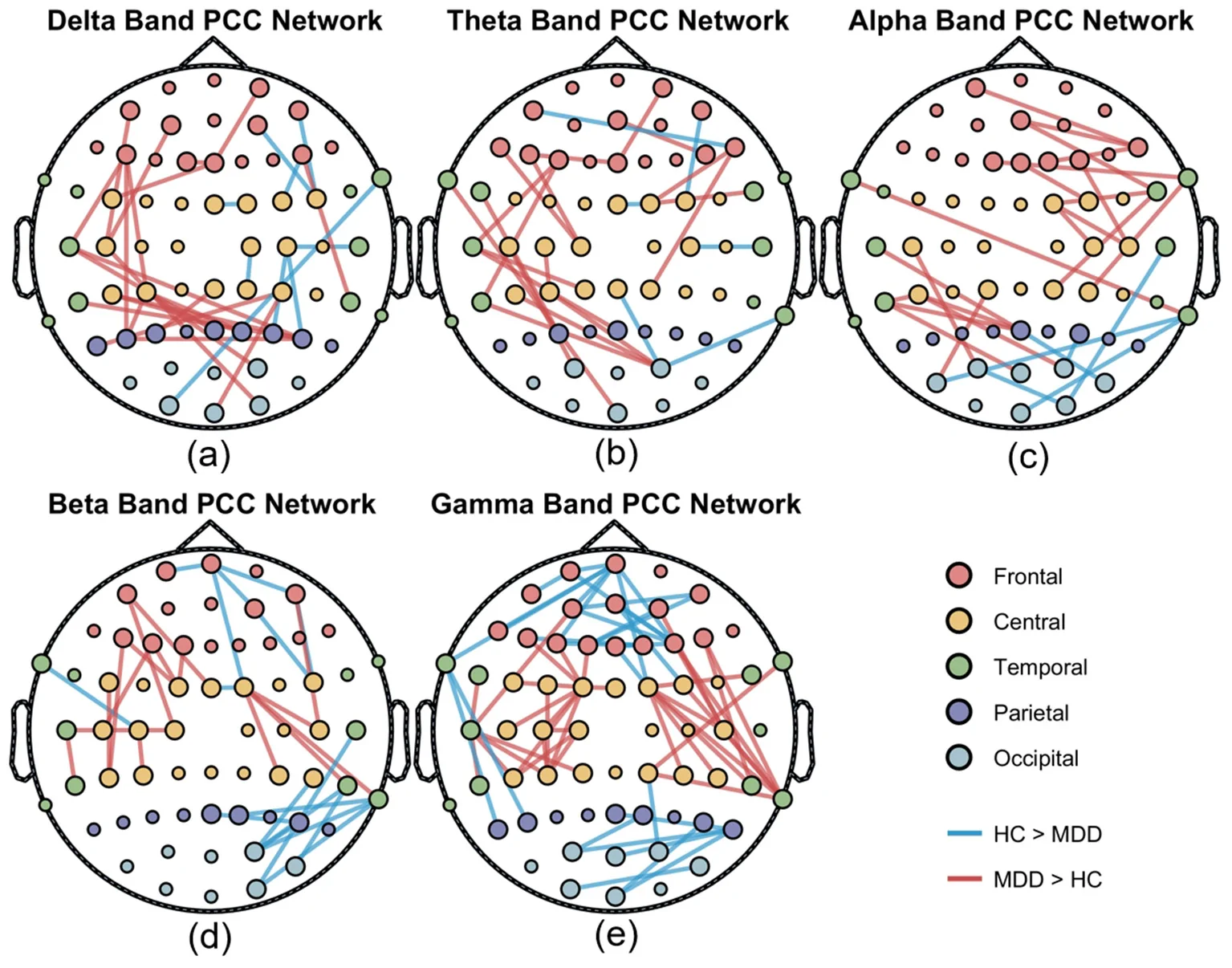
Statistical analysis of the PCC network, depicting significant connectivity edges (*p* < 0.05) across five frequency bands. **(a)** Delta band. **(b)** Theta band. **(c)** Alpha band. **(d)** Beta band. **(e)** Gamma band.

In the low-frequency bands, both PLV and PCC networks show enhanced connectivity in the left hemisphere. For the PLV network, enhanced connections in the δ and θ bands are mainly distributed among the left frontal, central, and parietal regions. In the θ band, stronger connections are observed between the frontal, parietal, and temporal regions. The PCC network exhibits a broader connectivity distribution. Enhanced correlations are observed not only in the left fronto-parietal regions but also in several long-range posterior connections. Overall, the low-frequency networks are characterized by increased left fronto-parietal connectivity and enhanced long-range connections. These alterations suggest that the spatial distribution and strength of functional interactions differ between individuals with MDD and HC in the low-frequency bands.

- **Mid-frequency bands (α** **and** **β** **bands)**: Increased fronto-central connectivity and reduced posterior connectivity

In the α and β bands, both PLV and PCC networks reflect increased connectivity involving the frontal and central regions, accompanied by reduced connectivity in posterior regions in the MDD group. In the PLV network, the α band exhibits increased connections mainly involving the frontal and central regions, with additional connections extending toward the temporal regions. In the β band, increased connections are primarily distributed among the frontal and central regions, whereas reduced connections are more evident in the parietal and occipital regions. The PCC network shows a similar pattern. Notably, the reduced connections in the parietal and occipital regions are more prominent in the β band. Overall, the α and β bands show a predominant pattern of increased fronto-central connectivity together with reduced posterior connectivity.

- **High-frequency band (γ** **band)**: Enhanced fronto-temporal and centro-temporal connectivity

In the γ band, both networks display increased connectivity involving frontal, central, and temporal regions and reduced connectivity in posterior regions in the MDD group. In the PLV network, increased connections are distributed across frontal-central, frontal-temporal, and central-temporal regions, while reduced connections are mainly observed among the parietal and occipital regions. The PCC network exhibits a similar pattern. Compared with the PLV network, the PCC network shows a broader distribution of altered connections involving these regions, particularly for connections between the temporal and posterior regions.

### 4.2 Significant node metrics

To further investigate the differences in local features within the network, we divided the 64 electrode nodes into 5 ROIs based on spatial distribution: frontal, central, temporal, parietal, and occipital regions. Figure 3 gives the regional divisions. BC, E_loc_, and CC were calculated for each ROI across five frequency bands. Subsequently, between-group statistical tests were performed to assess the significance of differences in these topological metrics, aiming to identify abnormal brain regions associated with MDD.

The network constructed through PLV displays centrality abnormalities in low-frequency bands, as demonstrated in Figure 7. The BC of the MDD group is significantly higher in the central regions than that of the HC group across the δ, θ, and α bands (*p* < 0.01), indicating increased centrality of these regions within the phase synchronization network. In contrast, in the α band, the MDD group shows significantly lower BC in the frontal and parietal regions compared with the HC group (*p* < 0.01).

**Figure 7:**
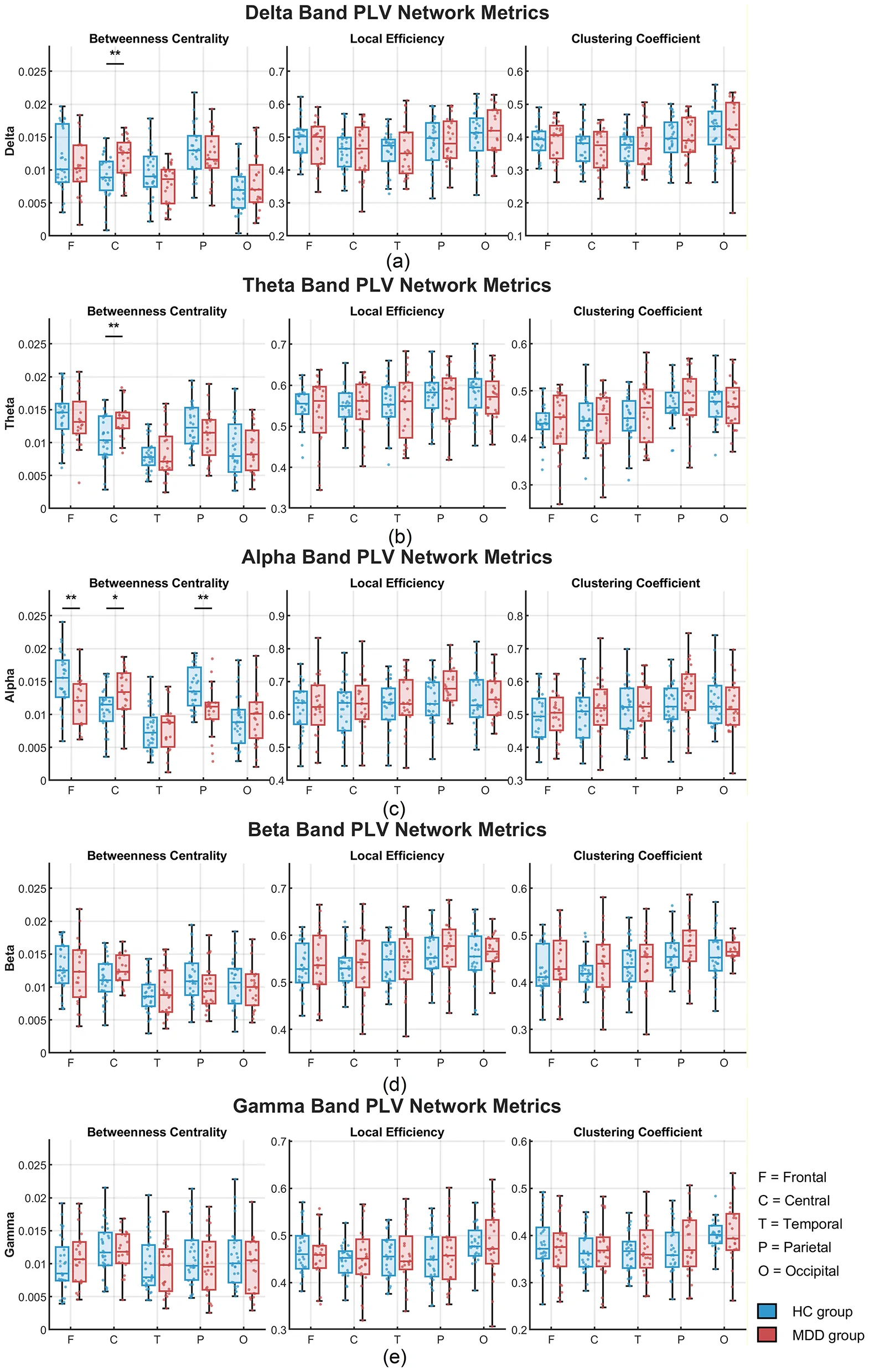
Statistical analysis of PLV network node metrics across five frequency bands and five brain regions. **(a)** Delta band. **(b)** Theta band. **(c)** Alpha band. **(d)** Beta band. **(e)** Gamma band. ^*^ indicates a significant between-group difference at *p* < 0.05, and ^**^ indicates a significant between-group difference at *p* < 0.01. The displayed significance levels are based on BH-FDR-adjusted results.

The differences between groups in the networks based on PCC are mainly concentrated in the parietal region of the α band, as expressed in Figure 8. The BC of the MDD group in the parietal region is significantly lower than that of the HC group (*p* < 0.01), with a similar downward trend observed in the β band. Additionally, in the α band, both *E*_loc_ and CC in the parietal region of the MDD group are higher than those of the HC group.

**Figure 8:**
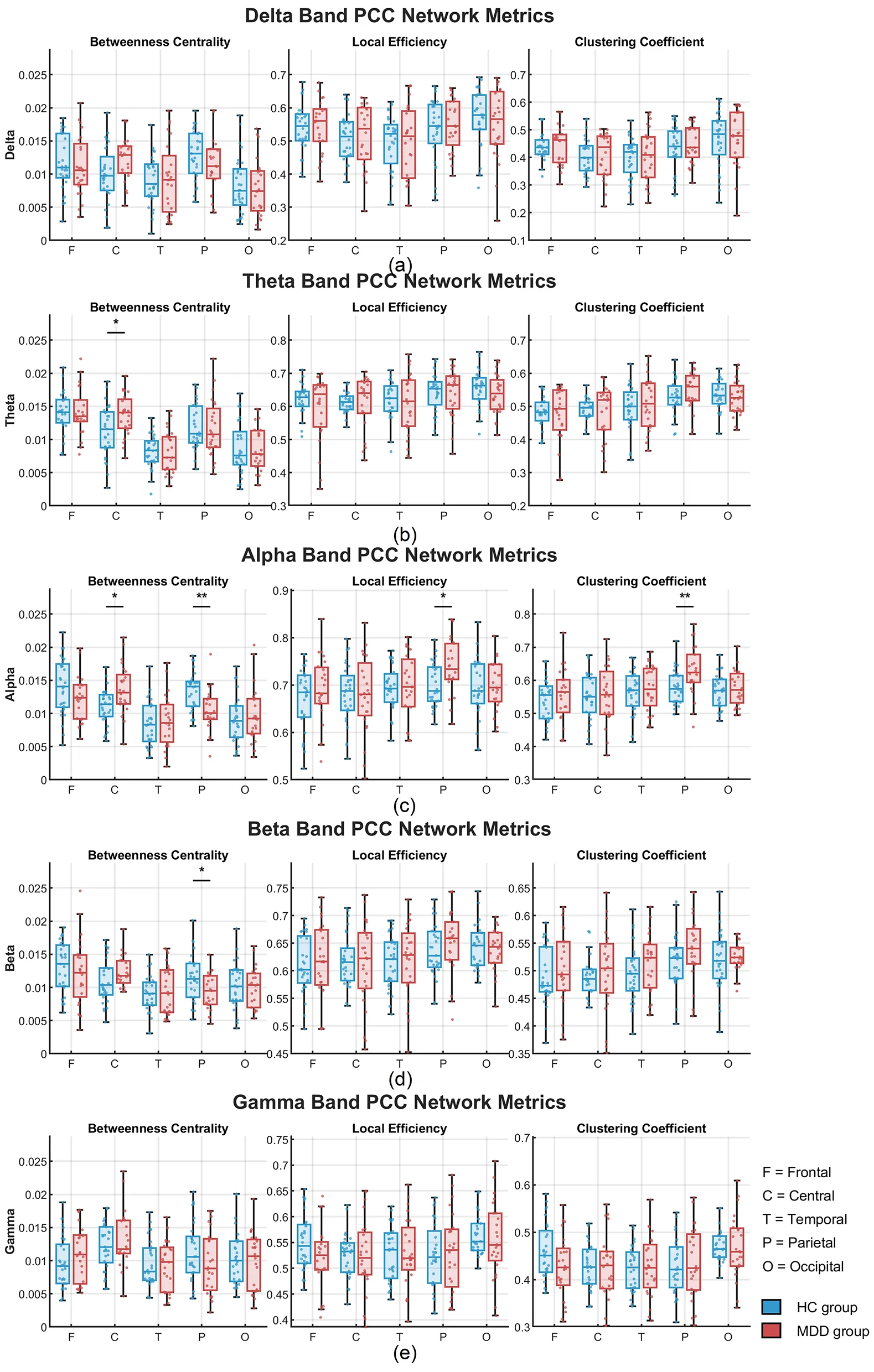
Statistical analysis of PCC network node metrics across five frequency bands and five brain regions. **(a)** Delta band. **(b)** Theta band. **(c)** Alpha band. **(d)** Beta band. **(e)** Gamma band. ^*^ indicates a significant between-group difference at *p* < 0.05, and ^**^ indicates a significant between-group difference at *p* < 0.01. The displayed significance levels are based on BH-FDR-adjusted results.

### 4.3 Classification performance

Compared to participant-level cross-validation performed only once, repeated participant-level cross-validation can be used to reduce randomness. In this study, we adopted a participant-level stratified repeated five-fold cross-validation strategy with five independent runs. Specifically, the 29 HC and 24 MDD subjects were evenly divided into 5 independent subsets according to the category ratio. In each cross-validation iteration, one subset was selected as the test set. In the remaining 4 subsets, one was further chosen as a validation set, while the other three were used for model training. All segments from the same participant belonged to the same set to ensure complete data independence. Importantly, statistical feature selection, normalization, hyperparameter optimization and statistical testing were performed exclusively within the training data within each fold.

SVM classification was performed on the extracted EEG segments, and the segment-level predictions were aggregated within each participant using a majority-voting strategy to obtain a participant-level prediction. The results presented in Tables 4–7 were obtained across five repeated runs of the cross-validation procedure. The SVM hyperparameter *C* was optimized utilizing the validation set over a predefined candidate range from 1 × 10^−4^ to 1 × 10^4^, including 15 candidate values. The five-fold cross-validation procedure was performed with a fixed random seed (random_seed = 42).

**Table 4:** Classification performance using PLV edge features.

Index	Repeat 1	Repeat 2	Repeat 3	Repeat 4	Repeat 5	Mean ±SD	95%CI
Accuracy (%)	77.36	75.47	73.58	77.36	73.58	75.47 ± 1.89	[73.12, 77.82]
Sensitivity (%)	66.67	75.00	70.83	70.83	70.83	70.83 ± 2.95	[67.18, 74.49]
Specificity (%)	86.21	75.86	75.86	82.76	75.86	79.31 ± 4.88	[73.25, 85.37]
Precision (%)	80.00	72.00	70.83	77.27	70.83	74.19 ± 4.20	[68.97, 79.40]
F1-score (%)	72.73	73.47	70.83	73.91	70.83	72.35 ± 1.45	[70.55, 74.16]
Balanced accuracy (%)	76.44	75.43	73.35	76.80	73.35	75.07 ± 1.65	[73.02, 77.12]
AUC (%)	86.85	80.89	85.20	87.72	82.54	84.64 ± 2.88	[81.07, 88.21]

**Table 5:** Classification performance using PCC edge features.

Index	Repeat 1	Repeat 2	Repeat 3	Repeat 4	Repeat 5	Mean ±SD	95%CI
Accuracy (%)	75.47	75.47	79.25	79.25	73.58	76.60 ± 2.54	[73.54, 79.68]
Sensitivity (%)	66.67	70.83	79.17	75.00	66.67	71.67 ± 5.43	[64.99, 78.35]
Specificity (%)	82.76	79.31	79.31	82.76	79.31	80.69 ± 1.89	[78.40, 82.98]
Precision (%)	76.19	73.91	76.00	78.26	72.73	75.42 ± 2.15	[72.84, 78.00]
F1-score (%)	71.11	72.34	77.55	76.60	69.57	73.43 ± 3.48	[69.30, 77.56]
Balanced accuracy (%)	74.71	75.07	79.24	78.88	72.99	76.18 ± 2.75	[72.97, 79.39]
AUC (%)	82.90	79.74	80.75	85.70	81.18	82.05 ± 2.34	[79.15, 84.95]

**Table 6:** Classification performance using PLV node features.

Index	Repeat 1	Repeat 2	Repeat 3	Repeat 4	Repeat 5	Mean ±SD	95%CI
Accuracy (%)	83.02	86.79	79.25	79.25	81.13	81.89 ± 3.15	[78.16, 85.62]
Sensitivity (%)	79.17	83.33	75.00	70.83	75.00	76.67 ± 4.75	[70.80, 82.54]
Specificity (%)	86.21	89.66	82.76	86.21	86.21	86.21 ± 2.44	[83.15, 89.27]
Precision (%)	82.61	86.96	78.26	80.95	81.82	82.12 ± 3.16	[78.16, 86.08]
F1-score (%)	80.85	85.11	76.60	75.56	78.38	79.30 ± 3.82	[76.64, 83.96]
Balanced accuracy (%)	82.69	86.49	78.88	78.52	80.60	81.44 ± 3.27	[77.18, 85.70]
AUC (%)	92.39	89.01	87.57	86.64	92.39	89.60 ± 2.68	[86.43, 92.77]

**Table 7:** Classification performance using PCC node features.

Index	Repeat 1	Repeat 2	Repeat 3	Repeat 4	Repeat 5	Mean ±SD	95%CI
Accuracy (%)	81.13	75.47	81.13	83.02	79.25	80.00 ± 2.86	[76.45, 83.55]
Sensitivity (%)	70.83	66.67	75.00	70.83	66.67	70.00 ± 3.48	[65.67, 74.33]
Specificity (%)	89.66	82.76	86.21	93.10	89.66	88.28 ± 3.93	[83.40, 93.16]
Precision (%)	85.00	76.19	81.82	89.47	84.21	83.34 ± 4.86	[77.30, 89.37]
F1-score (%)	77.27	71.11	78.26	79.07	74.42	76.03 ± 3.26	[71.98, 80.08]
Balanced accuracy (%)	80.24	74.72	80.61	81.97	78.16	79.14 ± 2.82	[75.63, 82.64]
AUC (%)	85.34	86.06	86.78	85.99	84.91	85.82 ± 0.72	[84.93, 86.71]

To evaluate the effectiveness of connectivity edge features in identifying MDD, PLV-Edge and PCC-Edge features were separately used as inputs to the SVM classifier. The results are presented in Tables 4, 5 where the 95% confidence intervals (CIs) were calculated using a one-sample t-distribution. For sensitivity, specificity, and precision, MDD was defined as the positive class. For PLV-Edge features, the mean balanced accuracy reaches 75.07%, with a mean specificity of 79.31%, indicating relatively stable discriminative performance across repeated cross-validation runs. For PCC-Edge features, the mean balanced accuracy is 76.18%.

For node features, PLV-Node and PCC-Node features were separately used for SVM classification, with the results summarized in Tables 6, 7. PLV-Node features achieve a mean balanced accuracy of 81.44%, alongside a mean sensitivity of 76.67% and mean specificity of 86.21%. PCC-Node features achieve a mean balanced accuracy of 79.14%, with a higher mean specificity of 88.28%. Compared with PCC-Node, PLV-Node shows higher accuracy with lower variability, suggesting more stable overall discriminative performance.

As displayed in Figure 9, the overall confusion matrices for PLV-Edge, PCC-Edge, PLV-Node, and PCC-Node features were obtained from five repeated five-fold cross-validation results. The PLV-Edge features achieve an MDD recognition rate of 70.83% and an HC recognition rate of 79.31%. For PCC-Edge features, the MDD recognition rate increases to 71.67%, while the HC recognition rate is 80.69%. The node-based features show slightly improved performance, with PLV-Node achieving recognition rates of 76.67% for MDD and 86.21% for HC, while PCC-Node achieves 70.00% and 88.28%, respectively.

**Figure 9:**
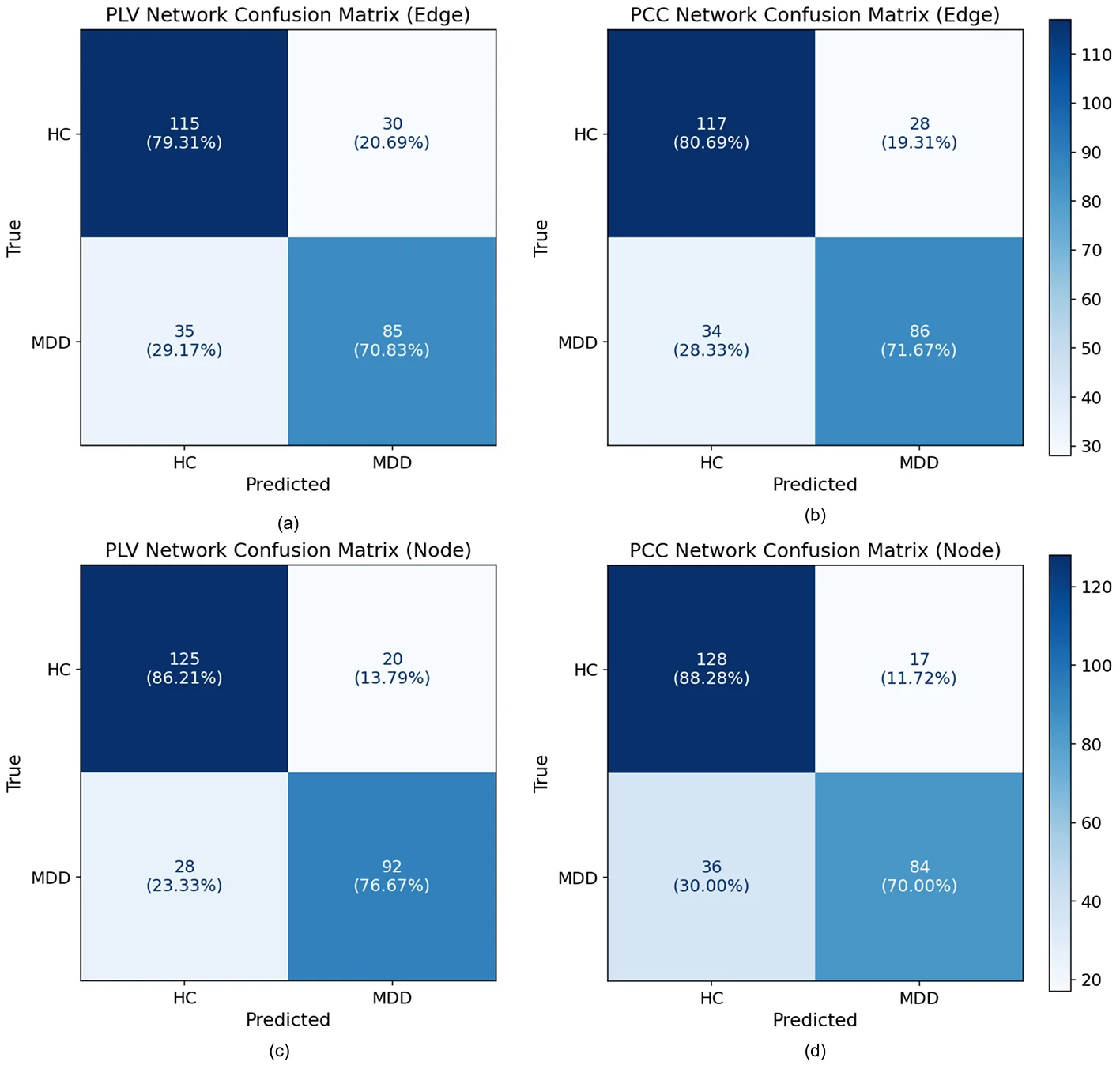
Overall confusion matrices based on four BFN features. **(a)** PLV-Edge; **(b)** PCC-Edge; **(c)** PLV-Node; **(d)** PCC-Node.

To further evaluate the classification performance of the proposed method, we compared the results with several existing EEG-based depression recognition studies (Earl et al., 2024; Wang et al., 2024; Li et al., 2025; Olbrich et al., 2026), as listed in Table 8. In our study, the SVM classifier is applied to four types of brain network features, namely PLV-Edge, PCC-Edge, PLV-Node, and PCC-Node features. Among these, PLV-Node features achieve the highest accuracy of 81.44% which is the highest among the controlled methods. These results suggest the advantage of our proposed method in EEG-based MDD identification.

**Table 8:** Comparison of existing mainstream with the proposed method.

Study	Feature	Classifier	Accuracy (%)
Earl et al., 2024	PLI functional connectivity features	Random Forest	60.00
Wang et al., 2024	PLV functional connectivity + spatio-temporal features	GCTNet	74.84
Li et al., 2025	PLI based source-level functional connectivity	SVM	76.20
Olbrich et al., 2026	Raw resting state EEG time series segments	Optimized 2D-CNN	67.50
Ours	PLV edge features	SVM	75.07
Ours	PCC edge features	SVM	76.18
Ours	PLV node features	SVM	**81.44**
Ours	PCC node features	SVM	79.14

The bold values indicate the accuracy rate of the controlled method and our proposed method that performed the best.

## 5 Discussion

This study constructs multi-frequency BFNs based on PLV and absolute PCC, revealing functional connectivity abnormalities and spatial distribution patterns in MDD across frequency bands. The results show that in the low-frequency bands, MDD patients exhibit increased functional connectivity in the left frontal, central, and parietal regions. In the mid-frequency bands, connectivity relatively increases in the frontal and central regions but declines in the posterior regions. In the high-frequency band, increased connectivity is mainly distributed among the frontal, central, and temporal regions, whereas reduced connectivity is primarily found in the posterior regions. These findings illustrate that MDD is not merely a local brain region abnormality but involves network reorganization across frequency bands and spatial scales. The observed patterns are consistent with previous EEG-based BFN studies. Previous studies have reported enhanced low-frequency synchronization in MDD, particularly in the frontal and central regions, which is associated with negative emotion processing and ruminative thinking (Wang et al., 2022). In the α band, hemispheric functional asymmetry has been widely documented: increased left frontal activity and decreased right posterior activity are considered typical electrophysiological features of MDD (Xie et al., 2023). In addition, reduced fronto-limbic connectivity in the γ band is consistent with the clinical signs in patients with mixed depressive features, such as impaired top-down emotion regulation and executive dysfunction (Xiong et al., 2026).

This study further reveals abnormal network characteristics of MDD at the node level in different brain regions and frequency bands. Results based on PLV show that MDD patients exhibit increased BC in the δ, θ, and α bands in the central region, and decreased BC in the α band in the frontal and parietal regions. Network abnormalities based on PCC are concentrated in the parietal region of the α band, showing decreased BC and increased *E*_loc_ and CC. These findings are consistent with previous studies. Previous reports have indicated that MDD patients often show increased centrality in regions related to sensorimotor functions, while the hub roles of the prefrontal and parietal cortex are weakened (Yang et al., 2023).

Based on these findings, we further explore the potential of BFN features in identifying MDD. Using the SVM classifier with five-fold cross-validation, four types of BFN features were evaluated. PLV-Node features achieve the highest mean balanced accuracy (81.44%). Overall, both edge- and node-level BFN features show discriminative potential for distinguishing MDD from HC, with PLV-Node features exhibiting the most favorable overall performance.

In the meantime, there are limitations as well. First things first, the sample size is relatively small, which may limit the generalizability to some extent. Moreover, the research was evaluated for binary classification between medication-free patients with clinically confirmed MDD and HC. As a result, the present findings do not support discrimination of depressive severity or other special circumstances like seasonal depression. The proposed biomarkers are applicable to the initial screening subjects who have been off medication for 2 weeks, and are not applicable to patients who are taking medication.

## 6 Conclusion

This study aims to investigate abnormal changes in BFN in MDD. BFNs were constructed from resting-state EEG data based on PLV and absolute PCC. A series of analyses were conducted on the BFNs. At the network connectivity level, MDD patients exhibit significant functional connectivity differences across multiple frequency bands. These differences include increased connectivity in the left hemisphere in the low-frequency bands, enhanced fronto-central connectivity and reduced posterior connectivity in the mid-frequency bands, and enhanced fronto-temporal and centro-temporal connectivity in the high-frequency band. At the node level, MDD patients show an altered distribution of hub regions. BC increases in the central regions across the δ, θ, and α bands. In the α band, BC falls in the frontal and parietal regions. The parietal region shows the most prominent alterations in the α band. In this region, BC decreases, whereas *E*_loc_ and CC enhance. These findings indicate frequency- and region-specific differences in regional network topology in MDD. The results further confirm the discrimination value of BFN features in identifying MDD. Among them, the PLV-Node features reach the highest average balanced accuracy of 81.44%. These findings suggest that BFN features capture complementary network information that may work in the objective identification of MDD.

## Data Availability

The MODMA dataset for this study can be found in the https://modma.lzu.edu.cn/data/index/. The original contributions presented in the study are included in the article, further inquiries can be directed to the corresponding author.
